# Dental Prosthesis

**Published:** 1912-02-15

**Authors:** 


### BIBLIOGRAPHY.

DENTAL PROSTHESIS. A manual of Dental Prosthesis, edited by George H. Wilson, of Cleveland, formerly Professor of Dental Prosthetics in the Dental Department of Western Reserve University has appeared from the well-known publishing house of Lea & Febiger of Philadelphia. The book is out of fashion for these days, in as much as it is written by a man actively working in the department of which he treats, instead of, as too many of our recent books being compiled by several writers. This is a one man book and it confines itself to one subject, and as might be expected it is sequential in treatment; and because of the character of the man, who writes it, there is much evidence of dogmatic statements which might be criticised were it not remembered that the author taught his subject for many years and learned that principles and dogmas have to be stated with earnestness to make them impressive to students and thoughtless readers. The book does not take up the entire subject of Dental Prosthesis, but confines its treatment to plate prosthesis entirely and this is one of the strong points in its favor. There is no attempt to introduce any part of crown and bridge work, or orthodontia, or even metallurgy. The author has been content to confine himself to plate work alone and this is perhaps a wise idea, although we should have preferred that a scientific treatment of the materials used in technical plate prosthesis might have had a place in the work. It would seem that every worker in dental prosthesis should have access in a work of this sort to a clear and intelligible statement of the materials involved as well as the technical processes. The order of treatment of the various subjects is systematic and the moderm methods are emphasized with sufficient clearness to make them intelligible with the aid of many well selected illustrations. The article on occlusion and articulation is well written, but we can't help wishing that some of the space given to the work of Gysi had been omitted, as it is confusing and will never become popular with the profession. Gysi has developed a few practical points in bite taking and occlusion that could be stated in simple terms that would be readily appreciated by anyone. But his articulating and bite frame are so complicated and expensive that no student will ever employ them, and few practitioners. The work is not a treatise, neither is it a compend, but is practically a manual that will be a most useful aid to every practitioner who wants to develop a good prosthetic technic with sufficient knowledge to enable him to make high technical and clinical attainments. The book is well illustrated and printed and makes a most valuable contribution to our literature. It shows much painstaking effort on the authors part and he is to be commended for devoting his time to so good a cause, especially since he is not now teaching and is therefore under no obligations to undertake work of this sort.

